# Spontaneous Sigmoid Colon Perforation and Ruptured Subserosal (“Zebra” Pattern) Small-Bowel Hematomas in Type IV Ehlers–Danlos Syndrome: A Case Report and a Short Review

**DOI:** 10.3390/jcm13144093

**Published:** 2024-07-12

**Authors:** Goran Augustin, Iva Radin, Tomislav Bubalo, Josip Mavrek, Goran Pavlek

**Affiliations:** 1 Department of Surgery, University Hospital Centre Zagreb, Kišpatićeva 12, 10000 Zagreb, Croatia; 2 School of Medicine, University of Zagreb, 10000 Zagreb, Croatia

**Keywords:** Ehlers–Danlos syndrome, colonic perforation, small-bowel hematoma

## Abstract

**Background and Objectives:** Spontaneous colonic perforations (SCPs) in teenagers and young adults are extremely rare. Common underlying conditions, such as colonic tumors and diverticulitis, are absent at that age. The vascular type of Ehlers–Danlos Syndrome (vEDS) is one cause of SCP. **Methods:** A 23-year-old male presented with an acute abdomen. The abdominal CT showed pneumoperitoneum with a large amount of fluid in the pelvis and abdomen, indicating hollow viscus rupture. At the level of the sigmoid colon, a defect in the intestinal wall and gas bubbles were seen. **Results:** Exploratory laparotomy confirmed sigmoid colon perforation without underlying pathology. Loop sigmoid colostomy was performed. Revisional surgery was undertaken due to clinical deterioration and intra-abdominal free fluid with small-bowel distension and air-liquid levels on abdominal CT 6 days later. Ileal subserosal hematomas were found, and many had ruptured, leaving a “zebra” pattern with lines of residual hematomas on the borders of subserosal hematomas. Genetic analysis confirmed vEDS. **Conclusions:** SCP in young adults or teenagers, in the absence of colonic disease, with clinical manifestations of connective tissue disorders should trigger genetic investigations for vEDS. SCP with a known vEDS could be treated with total colectomy to prevent further SCPs in the remaining colon. If segmental resections are performed, further SCP should be immediately excluded with any significant abdominal pain.

## 1. Introduction

Colonic perforation (Table 1) is a common complication of colorectal carcinoma (3–10%) or diverticulitis (22.5%) (Table 1) [1,2]. Colonic perforations can result from orally ingested foreign bodies. Approximately 80–90% of ingested foreign bodies spontaneously pass through the gastrointestinal tract. Less than 1% remain intraluminally, causing complications such as intestinal perforation [3]. Collagenous colitis is an extremely rare cause of colonic perforation [4].

On the other hand, colonic perforations in young adults are extremely rare due to the rarity of colorectal tumors, diverticulosis, or bowel ischemia. Even rarer are spontaneous colonic perforations (SCPs) at that age. Berry classified SCPs into stercoral and idiopathic [5]. Stercoral type is associated with chronic constipation; thus, it is rare in adolescents. The idiopathic type is sporadic and occurs at any age. It is a sudden perforation of the normal colon without underlying colonic pathology.

In this case report, we present a 23-year-old male with spontaneous perforation of the sigmoid colon with a non-specific medical history initially.

## 2. Case Report

**Figure 1:**
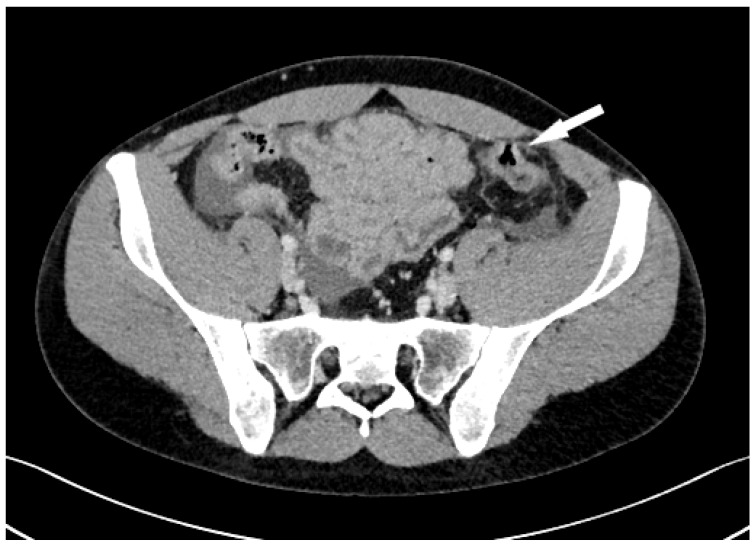
Abdominal CT shows sigmoid colon perforation (arrow).

**Figure 2:**
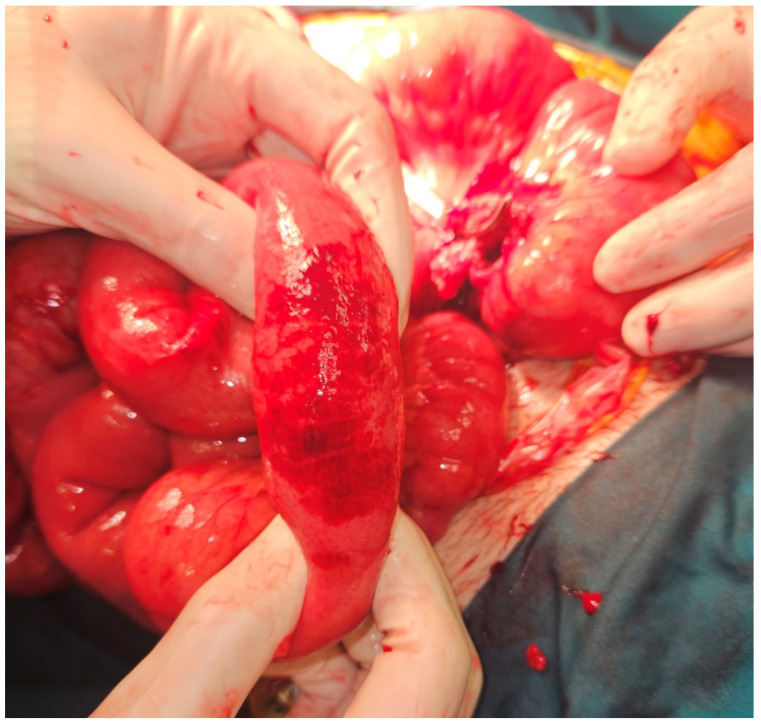
Ileal subserosal hematomas at revisional surgery (not present during the index operation).

**Figure 3:**
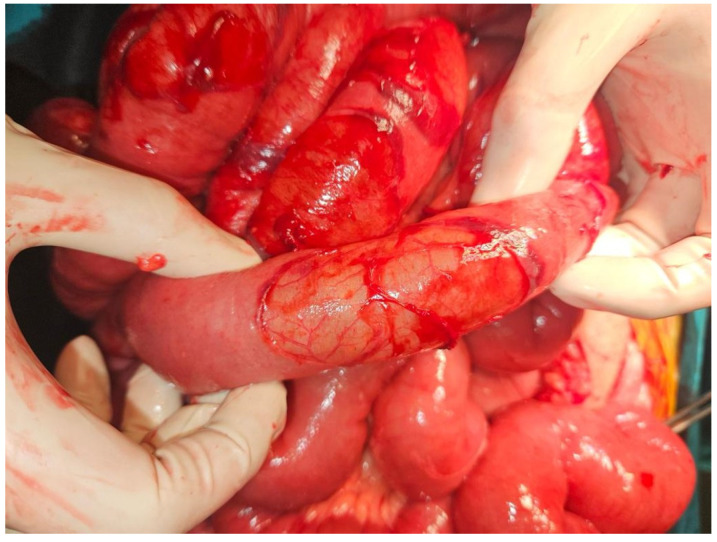
Ruptured hematomas have a “zebra pattern” with lines of residual hematomas on the borders of subserosal hematomas.

A 23-year-old male was admitted to the Emergency Department at University Hospital Centre Zagreb because of a sudden, cramp-like pain radiating from the back to the left inguinal region that started 20 min prior, without nausea, vomiting, or fever. Physical examination showed the abdomen at chest level, diffusely painful on palpation, with peritoneal irritation and muscle defense. Admission laboratory findings were as follows: erythrocytes 5.13 × 10^12^/L, hemoglobin 147 g/L, hematocrit 0.436 L/L, MCV 85 fL, thrombocytes 302 × 10^9^/L, leukocytes 17.5 × 10^9^/L, bilirubin 5 µmol/L, urea 3.4 mmol/L, creatinine 77 µmol/L, ALP 81 U/L, ALT 71 U/L, GGT 26 U/L, glucose 8.4 mmol/L, potassium 3.4 mmol/L, natrium 140 mmol/L, calcium 2.26 mmol/L, CRP < 1 mg/L, and GF-CDK 122 mL/min/1.73 m^2^. The electrocardiogram was normal, with a heart rate of 110 bpm. A plain abdominal X-ray was normal. Abdominal CT showed pneumoperitoneum with a large amount of fluid in the pelvis and abdomen, indicating hollow viscus rupture (Figure 1). At the transition to the sigmoid colon, a defect in the intestinal wall and gas bubbles were seen. The appendix was 1 cm long, without visible signs of acute appendicitis. The morphology of the abdominal parenchymal organs was normal. The biliary and urinary tract systems were not dilated. Bullous changes in the lung parenchyma were seen at the bases. Medical history revealed right-sided pneumothorax at the age of 15 and left-sided at 16 (both required VATS bullectomy), as well as bilateral inguinal hernia repair at the age of 3. The patient received 1 amp of trospium chloride, 1 amp of metoclopramide, and 100 mcg iv. fentanyl in the emergency department. The working diagnosis was an acute abdomen from the sigmoid colon perforation of unknown cause. Emergent median laparotomy showed abundant intestinal contents in all quadrants. After extensive lavage, sigmoid colon perforation was confirmed, and a loop sigmoid colostomy at the perforation site was made. On the sixth postoperative day, the patient became febrile, and CRP values increased. Abdominal CT showed a large amount of intraabdominal free fluid with small-bowel distension and air-liquid levels, so reoperation was indicated. At relaparotomy, distended small-bowel loops were found in the left lower quadrant, which had abundant clear free fluid. The appendix looked macroscopically changed, probably secondary. Lavage, appendectomy, and terminal ileum resection with end ileostomy were conducted. Interestingly, ileal subserosal hematomas (Figure 2) were found. Many of these ruptured, leaving a “zebra pattern” with lines of residual hematomas on the borders of subserosal hematomas (Figure 3).

**Figure 4:**
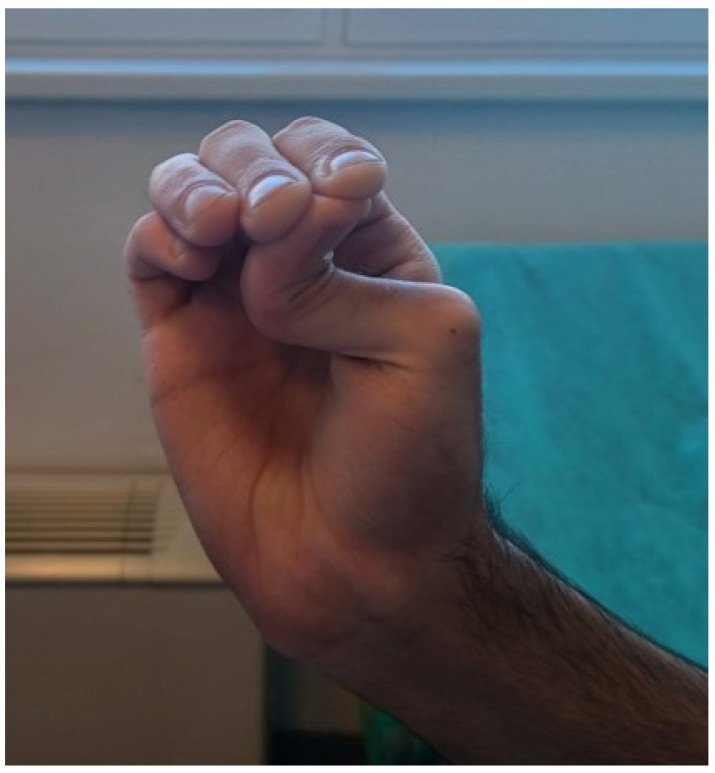
Small joint hypermobility—one of the “minor” criteria for Ehlers–Danlos syndrome.

**Figure 5:**
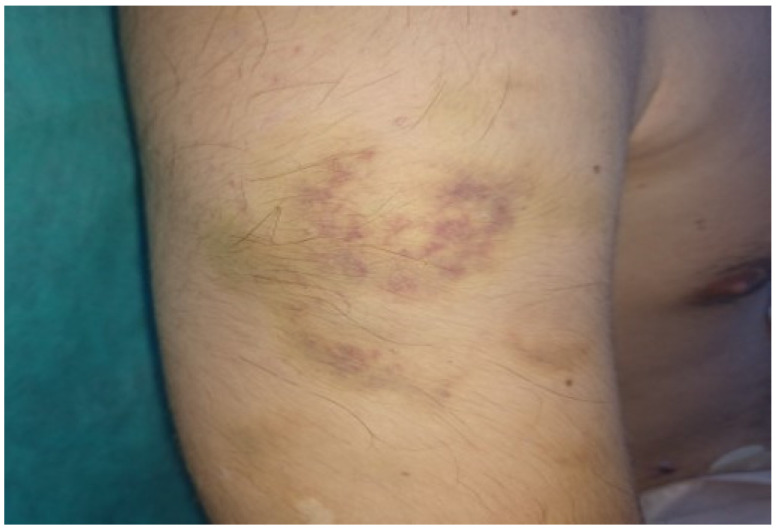
Easy bruising—one of the “minor” criteria for Ehlers–Danlos syndrome.

Because of spontaneous colonic perforation, skin hyperelasticity, joint hypermobility (Figure 4), easy bruising (Figure 5), two spontaneous pneumothoraces, centrilobular emphysema, and lung bullae in the medical history, the diagnosis of Ehlers–Danlos syndrome (EDS) was suspected. Furthermore, gene sequencing showed mutations in the collagen type III alpha 1 chain (COL3A1) gene (position 2:189853361, DNA c.628G > C, protein p.Gly210Arg, HET genotype). This mutation confirms EDS type IV (vascular type—vEDS). The patient was examined by an immunologist and rheumatologist (suspected vasculitis), a hematologist (suspected pulmonary embolism), a medical genetics specialist (suspected EDS), an ophthalmologist (ophthalmological complications of EDS), and a cardiologist (cardiovascular complications of EDS—aneurysms, dissection). The patient was discharged two weeks after the second operation and had no other gastrointestinal complications five months later. On follow-up, one month after discharge, a cardiologist prescribed 200 mg of celiprolol (selective β_1_ receptor antagonist and β_2_ receptor partial agonist) to reduce the chances of aortal dissection and rupture and 1500 mg of ascorbic acid daily, which is important for collagen synthesis and also has antioxidant properties.

## 3. Discussion

EDS represents a group of inherited connective tissue disorders. Mutations in collagen result in the defective function of tissues and organs, mostly skin, joints, and blood vessel walls. In 2017, the International EDS Consortium divided EDS into 13 subtypes [6]. vEDS is uncommon (3–6% of all EDS cases), but it is the most severe type. It is an autosomal dominant form, with an estimated prevalence of 1/50,000 to 1/20,000 [7]. It results from different mutations in the COL3A1 gene encoding collagen type III, rarely COL1A1 (collagen type I). Two aspects of vEDS related to gastrointestinal (GI) complications are found in our case and further discussed.

## 4. Spontaneous Colonic Perforation

The prevalence of specific connective tissue disorder (CTD) etiologies does not correspond to the incidence of spontaneous intestinal perforations (SIPs). The most prevalent of the included CTDs in the general population is rheumatoid arthritis (RA), with a prevalence of 500/100,000, followed by systemic lupus erythematosus (SLE) (73/100,000), systemic sclerosis (SS) (7-34/100,000), polyarteritis nodosa (PN) (3.1/100,000), and granulomatosis with polyangiitis (GPA) (2.4/100,000). The prevalence of Churg–Straus syndrome (CS) and type IV EDS is much lower, at 1.1/100,000 and 1/100,000, respectively.

The descending incidence of SIPs is 13% with PN [8,9], 10,7% with vEDS [10,11], and 9% with CS [9], although the CS sample is very small (1/11 patients). More cases of SIPs from CS are found mostly in the Japanese literature [9,12]. Further in descending order is SLE (3.6%) [13,14]. Interestingly, although SLE involves the GI tract less commonly than SS, it results in SIPs more commonly (rectum 50%—caused by lupus mesenteric vasculitis; colon 27.7%; ileum 16.7%) [15]. SIPs are the least common with RA (0.09%) [9,16]. We could not determine the incidence of SIPs from GPA, formerly Wegener granulomatosis, and SS because they were only reported as individual cases. Interestingly, 80% of SS patients have lower GI tract involvement, and 20–50% are symptomatic in the form of constipation or diarrhea. There is no association between gut involvement and specific SS subtypes [17]. Our search results are limited by the rarity of published cases and the lack of databases with larger numbers of patients with different CTDs. Table 2 shows the available results.

SCP is the single most common GI complication of vEDS [25]. Most spontaneous GI perforations in vEDS occur in the sigmoid colon, followed by the small bowel, while gastric perforations are exceptional [11]. GI perforations are significantly more common in men [11]. The first SCPs start in the late teenage years, moving into the late twenties, and most precede the molecular diagnosis of vEDS [25,26], as in our case. There are cases of SCPs at more advanced ages, which can result from different mutations with different clinical vEDS severities [27].

The pathophysiology of SCP depends on the type of CTD. In SS, colonic wall fibrosis causes progressive constipation and colonic wall sacculations with impacted feces and resultant SCP [28]. Changes in the colonic microbiome and an increased rate of infections could increase SCP risk [29], especially with corticosteroid therapy [24]. The pathophysiology is partly responsible for the age of presentation. SS-caused SCP presents in the fifth decade or later [20,24].

Although no specific therapy for vEDS exists, blood-lowering medications, such as celiprolol (selective β1 receptor antagonist and β2 receptor partial agonist), reduce heart rate, mean, and pulsatile pressures and may have a protective effect in vEDS [30]. Ascorbic acid at a 500 mg daily dose reduces the risk of aortal dissection and rupture [31].

Hartmann’s procedure is the most common procedure in the management of SCP in vEDS, especially if the underlying vEDS is unknown at the event of SCP. Other procedures include colectomy with primary anastomosis, the creation of colostomy by exteriorizing the perforation site, the primary closure of the perforation with or without a protective ileostomy, and total colectomy with a permanent ileostomy. The incidence of postoperative complications is high, including intra-abdominal bleeding, wound dehiscence, enterocutaneous fistulas, and adhesions. 

Half (47%) of patients with an SCP are at risk of early reperforation in the remaining colon within 20 months in the absence of adequate therapeutic management [25]. Considering the high rate of reperforations, some recommend total abdominal colectomy with end ileostomy or ileorectal anastomosis [25,32,33] when vEDS is confirmed. An additional risk factor for perforation is male sex, so elective total colectomy after emergent segmental resection when vEDS is confirmed could be offered to this subgroup.

A retrospective study by Sugimoto et al. demonstrated that cumulative survival in CTD patients is significantly worse than without CTD after surgery for colorectal perforation [34]. There were no patients with EDS. The first patient that survived after treatment of SCP from SS was reported in 1972 [20].

## 5. Spontaneous Small-Bowel Hematomas

The second aspect is spontaneous intramural small-bowel hematomas, which have been rarely described. A single-center study found that all patients had a risk factor for bleeding—oversaturation with warfarin, hemophilia, liver failure after chemotherapy, cirrhosis, systemic lupus vasculitis, and idiopathic thrombocytopenic purpura [35]. We were unable to find a reported case of multiple mesenteric lacerations and ruptured subserosal hematomas of the small bowel as the postoperative complication after surgery for SCP in type IV EDS.

Although the exact mechanism is unknown, we hypothesize that the defect in type III collagen, in addition to a decreased intima-media thickness, results in increased mechanical stress applied to excessively fragile tissues [36]. This increases the risk of arterial dissection or rupture. High small-bowel intraluminal pressure, possibly from paresis (peritonitis plus operation), forceful peristalsis after initial paresis, and intraoperative small-bowel manipulation, caused spontaneous subserosal hematomas, some of which ruptured and were observed during the revisional operation. The gross appearance of centrally ruptured subserosal small-bowel hematomas, with the remaining undrained hematomas on the edges, had an appearance of a “zebra” pattern.

## 6. Conclusions

SCPs are uncommon in the general population. Colonic perforations typically result from diverticulitis or colonic carcinoma in people in their fifties or later. Therefore, SCP in young adults or teenagers, in the absence of known colonic disease, especially if CTD clinical manifestations are obvious, should trigger genetic investigations for vEDS. SCP in males with a known vEDS could be treated with total colectomy to prevent further SCPs in the remaining colon. If segmental resections are performed, further SCPs should be immediately excluded with any significant abdominal pain.

The case was presented at the Seventh Emergency Medicine Congress with international participation, 25–28 April 2024, Pula, Croatia.

## Figures and Tables

**Table 1:** Common causes of colonic perforation.

Category	Causes
Inflammatory	Colonic diverticulitis Inflammatory bowel disease Endometriosis *
Tumors	Colorectal cancer Colon perforation from obstruction by distal colon cancer or extracolonic tumor
Vascular	Mesenteric ischemia Ischemic colitis Connective tissue disorders
Non-neoplastic obstruction	Volvulus Enterolith Colonic gallstone ileus Endometriosis Fecal impaction
Other	Colonic pseudo-obstruction Intraluminal foreign bodies
Trauma	
Intervention	Colonoscopy

* endometriosis is not solely an inflammatory cause; it can be party obstructive.

**Table 2:** Incidence and the rate of spontaneous colonic perforations in various connective tissue diseases.

Connective Tissue Disease	Study	No. of Patients	No. of Perforations	Incidence of Perforations (%)	Prevalence in the General Population
Polyarteritis nodosa	Levine et al. [8]	54	6	13%	3.1/100,000
	Pagnoux et al. [9]	38	6		
Ehlers–Danlos type IV	Pepin et al. [11]	419	41	10.67%	1/100,000
	Oderich et al. [10]	31	7		
Churg–Straus syndrome	Pagnoux et al. [9]	11	1	9%	1.1/100,000
Systemic lupus erythematosus	Zizic et al. [13]	107	5	3.6%	73/100,000
	Pombo et al. [14]	220	7		
Rheumatoid arthritis	Curtis et al. [16]	40,841	37	0.09%	500/100,000
	Pagnoux et al. [9]	3	1		
Granulomatosis with polyangiitis	McNabb et al. [18]	Individual cases	1		2.4/100,000
	Geraghty et al. [19]		1		
Systemic sclerosis	Jayson et al. [20]	Individual cases	1		7-34/100,000
	Battle et al. [21]		1		
	Ebert et al. [22]		1		
	Guan et al. [23]		1		
	Stupalkowska et al. [24]		1		

## Data Availability

Electronic medical record at University Hospital Centre Zagreb.
